# SARS-CoV-2 seroprevalence and infection rate in Manila, Philippines prior to national vaccination program implementation: a repeated cross-sectional analysis

**DOI:** 10.1186/s41182-022-00468-7

**Published:** 2022-10-11

**Authors:** Greco Mark B. Malijan, Tansy Edwards, Kristal An Agrupis, Shuichi Suzuki, Annavi Marie G. Villanueva, Ana Ria Sayo, Ferdinand De Guzman, Alexis Q. Dimapilis, Rontgene M. Solante, Elizabeth O. Telan, Dorcas V. Umipig, Kenji Ota, Fumitaka Nishimura, Katsunori Yanagihara, Mary Jane Salazar, Edmundo B. Lopez, Koya Ariyoshi, Chris Smith

**Affiliations:** 1 San Lazaro Hospital-Nagasaki University Collaborative Research Office, San Lazaro Hospital, Quiricada St., Sta. Cruz, 1003 Manila, Philippines; 2 School of Tropical Medicine and Global Health, Nagasaki University, Sakamoto, Nagasaki, 852-8102 Japan; 3 MRC International Statistics and Epidemiology Group, London School of Hygiene & Tropical Medicine, London, WC1E 7HT UK; 4 San Lazaro Hospital, Quiricada St., Sta. Cruz, 1003 Manila, Philippines; 5 Department of Laboratory Medicine, Nagasaki University Hospital, Sakamoto, Nagasaki, 852-8102 Japan; 6 Institute of Tropical Medicine, Nagasaki University, Sakamoto, Nagasaki, Nagasaki 852-8523 Japan; 7 Department of Clinical Research, London School of Hygiene & Tropical Medicine, London, WC1E 7HT UK

**Keywords:** SARS-CoV-2, COVID-19, Seroepidemiological study, Philippines

## Abstract

**Background:**

SARS-CoV-2 seroepidemiological studies are used to guide public health decision making and to prepare for emerging infectious diseases. Disease occurrence estimates are limited in the Philippines, the country with the highest reported number of coronavirus disease-related deaths in the Western Pacific region. We aimed to estimate SARS-CoV-2 seroprevalence and infection rate among outpatient clinic attendees in Metro Manila prior to the implementation of the national coronavirus disease vaccination program.

**Methods:**

We conducted repeated cross-sectional surveys at the animal bite clinic in San Lazaro Hospital, Manila, the Philippines across four periods, 3 months apart, between May 2020 and March 2021. Multivariable logistic regression was used to assess associations between different characteristics and infection status including seropositivity.

**Results:**

In total 615 participants were enrolled, ranging from 115 to 174 per period. Seroprevalence quadrupled between the first (11.3%) and second (46.8%) periods and plateaued thereafter (third—46.0%, fourth—44.6%). Among seropositive participants, total antibody concentration was comparable throughout the first to third periods but declined between the third and fourth periods. Infection prevalence was comparable across enrollment periods (range 2.9–9.5%). Post-secondary education [aOR 0.42 (95% CI 0.26, 0.67)] was protective, and frontline work [aOR 1.81 (95% CI 1.18, 2.80)] was associated with increased odds of seropositivity. Frontline work status [aOR 2.27 (95% CI 1.10, 4.75)] and large household size [aOR 2.45 (95% CI 1.18, 5.49)] were associated with increased odds of infection.

**Conclusions:**

The quadrupling of seroprevalence over 3 months between the first and second enrollment periods coincided with the high burden of infection in Metro Manila in early 2020. Our findings suggest a limit to the rise and potential decline of population-level SARS-CoV-2 infection-induced immunity without introduction of vaccines. These results may add to our understanding of how immunity develops against emerging infectious diseases including coronaviruses.

## Background

On March 2020 the Philippines was placed under enhanced community quarantine, heralding one of the longest and most stringent lockdowns during the global coronavirus disease (COVID-19) pandemic [1]. The securitized response to the pandemic involved international and subnational border restrictions, closure of all but essential establishments and services, cessation of mass public transport, stay at home orders, and stringent policies on personal protective equipment use [2]. Despite these containment measures, the country reported the highest number of cumulative COVID-19-related cases and deaths in the Western Pacific region by December 2021 [3].

Serological tests detect presence of past and/or current infection, and seroepidemiological studies are used to estimate actual COVID-19 prevalence prior to the introduction of mass immunization. Current evidence suggests that antibodies recognizing SARS-CoV-2 peak 2–3 weeks after disease onset and may remain detectable up to 6–12 months [4]. Early in the pandemic, sero-epidemiological investigations were used to study COVID-19 burden and transmission and to guide allocation of limited resources including vaccines [5]. Global seroprevalence by December 2020 was estimated to be low in the general population (median 4.5%), but significant heterogeneity was seen across subpopulations, from perinatal women (0.6%) to persons in assisted living facilities (59.0%) [6].

Seroepidemiological studies have focused on specific populations, such as blood donors [7], cancer patients [8], and healthcare workers [9]. Little is known about the seroprevalence and consequent changes through time in the Philippines. We conducted repeated cross-sectional surveys to estimate SARS-CoV-2 seroprevalence and infection rate among patients and contacts attending an outpatient animal bite clinic, as surrogates for the catchment population, in a tertiary infectious disease referral hospital in Metro Manila, the Philippines.

## Methods

### Study design

This repeated cross-sectional analysis is part of a larger Acute Respiratory Tract Infection (ARI) study, aimed at describing the epidemiology and clinical features of ARI among patients, healthcare workers (HCW), and household contacts in San Lazaro Hospital (SLH) in Metro Manila, the Philippines.

### Setting

Situated in the Outpatient Department, the animal bite clinic (ABC) provides rabies post-exposure prophylaxis for all, free at point of use through the national health insurance system. Most clinic attendees come from Metro Manila. SLH–ABC is one of the largest animal bite treatment centers in the country, attending to an average of 200 new patients daily. While other similar centers reduced their operations or closed during lockdown periods [10, 11], SLH–ABC continued its operations.

**Fig. 1:**
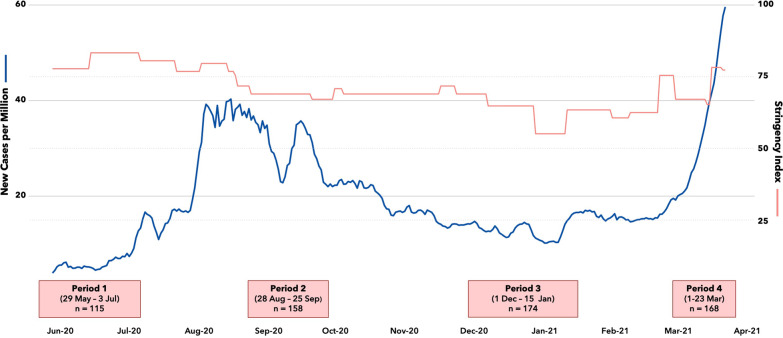
Data collection flow. Study enrollment took place over four periods, during which time the Philippines underwent varying levels of lockdown. The Oxford COVID-19 Government Response Tracker Stringency Index aggregates and summarizes the closure and containment policy indicators employed at national and subnational levels in response to the pandemic. It takes values between 0 and 100, with the most stringent government response set at 100

Enrollment took place over four periods, roughly 3 months apart: 29 May to 3 July 2020; 28 August to 25 September 2020; 1 December 2020 to 15 January 2021; and 1–23 March 2021. Metro Manila underwent varying levels of lockdown during the study (Fig. 1). In the first enrollment period, non-essential workers were prohibited from travelling outside their households. Throughout the second to fourth enrollment periods, most individuals, except the clinically vulnerable, elderly, and children, were permitted movement outside their households. In addition, minors aged 17 and under were given stay-at-home orders during the third enrollment period [12]. The second enrollment period coincided with the downward trajectory of the first COVID-19 wave, and the fourth enrollment period took place on an upward trajectory of the second COVID-19 wave in the country [13]. Vaccination rollout began in March 2021 for HCWs and in May 2021 for non-HCWs [14].

### Participants

New patients consulting at ABC and/or their household contacts greater than 1 year of age were eligible for enrollment. In each data collection period, research nurses approached clinic attendees in the dedicated waiting areas. ABC attendees were invited to participate, and those who provided written informed consent were enrolled consecutively. There were no limitations on daily recruitment.

### Outcomes

Seropositivity was assessed using Elecsys Anti-SARS-CoV-2 immunoassay (Roche Diagnostics, Basel, Switzerland), with 99.5% sensitivity and 99.8% specificity, based on studies involving symptomatic COVID-19 patients [15, 16]. The cutoff index for a reactive test is set at ≥ 1 AU/mL. The test measures total SARS-CoV-2 antibodies including IgG, IgA, and IgM, but it does not return immunoglobulin class-specific results [17].

COVID-19 infection was confirmed using real-time polymerase chain reaction (RT-PCR) detecting RdRP and E genes from extracted viral RNA (Qiagen Viral RNA Mini Kit, Hilden, Germany) from nasopharyngeal and oropharyngeal swab specimens using Corman et al. [18] and Nao et al. [19] protocols in StepOnePlus Real-Time PCR system (Applied Biosystems, Massachusetts, United States).

We operationally defined seroprevalence as the proportion of the population who have antibodies against SARS-CoV-2 and the infection rate as the proportion of the population with detectable SARS-CoV-2 RNA on RT-PCR.

### Other data

Participant demographics, socioeconomic information, medical history, COVID-19 exposure history, personal protective practices, and clinical symptoms were collected via questionnaire interview. To categorize exposure risks, reported participant occupations were categorized into frontline essential and non-frontline essential work status based on the Department of Health (DOH) vaccination priority framework [14]. Frontline workers included healthcare workers, uniformed personnel, sanitary personnel, drivers, delivery and logistics personnel, food production and grocery staff, manual laborers, security personnel, formal and informal vendors, wellness staff, and government workers in justice, security, transport, and social protection sectors.

Interview data were collected and stored electronically through REDCap [20]. RT-PCR and serology results were provided via Microsoft Excel files [21] then entered and stored electronically through REDCap.

### Sample size

A minimum sample size of 100 individuals per enrolment period would allow estimation of seroprevalence at least 15% with 10% absolute precision.

### Statistical methods

We summarized participant characteristics (demographics, medical and exposure history, personal protective practices, presence of COVID-19 symptoms, SARS-CoV-2 positivity and seropositivity), stratified by data collection period. Continuous data were expressed as mean (SD) and median (IQR), and categorical data were expressed as number (%). Chi-squared test and one-way analysis of variance were used to assess differences in participant characteristics across the four periods. Infection rate and seroprevalence were reported with 95% binomial confidence intervals. To investigate associations between participant characteristics and SARS-CoV-2 infection and seropositivity, we used logistic regression. Characteristics associated with the outcome in univariable analyses based on a likelihood ratio test (LRT) *p* value < 0.1 were considered for inclusion in stepwise multivariable model building and retained if the LRT with and without the characteristic in an adjusted, final model had *p* value < 0.05. We included sex, age group, and enrollment period a priori during model building. Data cleaning, analysis, and visualization were performed in R and RStudio [22].

## Results

### Participants

In total, 615 individual participants were enrolled (range 115–174 participants per period). Table 1 shows the characteristics of enrolled participants. Overall, approximately half (55.6%) of the participants were female; 86.0% were adults of working age; 4.7% were older adults (60 years and above), and 9.3% were children (< 18 years). Amongst 558 adults, 36.4% were frontline workers and 73.7% had post-secondary education. Of 57 participants aged < 18 years, two (3.5%) reported working as a general goods or *sari-sari* store vendor and were classified as frontline workers, and five (8.8%) reported attaining post-secondary education. Monthly household income data were available for 471 (76.6%) participants, including 32 (56.1%) of 57 child participants.

**Table 1:** Participant characteristics stratified by data collection period

	Period 1 29 May 2020 to 3 Jul 2020 *n* = 115 (%)	Period 2 28 Aug 2020 to 25 Sep 2020 *n* = 158 (%)	Period 3 1 Dec 2020 to 15 Jan 2021 *n* = 174 (%)	Period 4 1 Mar 2021 to 23 Mar 2021 *n* = 168 (%)	Total *N* = 615 (%)	*p* value
Sex						
Female	62 (53.9)	100 (63.3)	99 (56.9)	81 (48.2)	342 (55.6)	0.052
Male	53 (46.1)	58 (36.7)	75 (43.1)	87 (51.8)	273 (44.4)	
Age						
Mean (SD)	35.2 (15.2)	36.4 (14.0)	36.6 (14.3)	33.9 (13.8)	35.5 (14.3)	0.292
Median [IQR]	32.0 [23.2, 43.2]	34.0 [24.1, 47.5]	35.9 [26.5, 45.6]	32.8 [22.5, 42.4]	33.5 [24.0, 45.3]	
Age groups						
Below 18	10 (8.7)	10 (6.3)	14 (8.0)	23 (13.7)	57 (9.3)	0.366
18–39	63 (54.8)	86 (54.4)	95 (54.6)	91 (54.2)	335 (54.5)	
40–49	23 (20.0)	35 (22.2)	29 (16.7)	27 (16.1)	114 (18.5)	
50–59	12 (10.4)	17 (10.8)	29 (16.7)	22 (13.1)	80 (13.0)	
60 and above	7 (6.1)	10 (6.3)	7 (4.0)	5 (3.0)	29 (4.7)	
Education^a^						
None	2 (1.7)	0	0	0	2 (0.3)	**0.001**
Primary	11 (9.6)	7 (4.5)	15 (8.7)	12 (7.3)	45 (7.4)	
Secondary	28 (24.3)	48 (30.6)	37 (21.4)	24 (20.6)	147 (24.1)	
Vocational	5 (4.3)	16 (10.2)	6 (3.5)	26 (15.8)	53 (8.6)	
Tertiary	68 (59.1)	86 (54.8)	114 (65.9)	91 (55.2)	359 (58.9)	
Post-graduate	1 (0.9)	0	1 (0.6)	2 (1.2)	4 (0.7)	
Occupation^a^						
Call center agent	4 (3.5)	7 (4.4)	2 (1.1)	1 (0.6)	14 (2.3)	**0.001**
Cleaning services	1 (0.9)	12 (7.6)	1 (0.6)	20 (12.4)	34 (5.6)	
Delivery services	0	1 (0.6)	3 (1.7)	2 (1.2)	6 (1.0)	
Driver	1 (0.9)	6 (3.8)	8 (4.6)	6 (3.7)	21 (3.5)	
Factory worker	1 (0.9)	1 (0.6)	0	0	2 (0.3)	
Food services	4 (3.5)	7 (4.4)	1 (0.6)	2 (1.2)	14 (2.3)	
Grocery staff	10 (8.8)	2 (1.3)	4 (2.3)	3 (1.9)	19 (3.1)	
Healthcare worker	0	3 (1.9)	2 (1.1)	4 (2.5)	9 (1.5)	
House helper	2 (1.8)	1 (0.6)	3 (1.7)	0	6 (1.0)	
Laborer	8 (7.0)	8 (5.1)	16 (9.2)	10 (6.2)	42 (6.9)	
Office worker	12 (10.5)	13 (8.2)	19 (10.9)	12 (7.5)	56 (9.2)	
Overseas Filipino worker	0	1 (0.6)	1 (0.6)	0	2 (0.3)	
Others	18 (15.8)	19 (12.0)	23 (13.2)	21 (13.0)	81 (13.3)	
Security personnel	2 (1.8)	0	5 (2.9)	3 (1.9)	10 (1.6)	
Vendor	3 (2.6)	7 (4.4)	8 (4.6)	6 (3.6)	24 (4.0)	
Wellness or grooming	1 (0.9)	0	0	1 (0.6)	2 (0.3)	
Unemployed	47 (41.2)	70 (44.3)	78 (44.8)	70 (43.5)	265 (43.7)	
Frontline essential worker^a,b^						
Yes	31 (32.0)	53 (34.0)	54 (31.2)	67 (41.1)	205 (34.8)	0.238
No	66 (68.0)	103 (66.0)	119 (68.8)	96 (58.9)	384 (65.2)	
Monthly household income (PHP)^a^						
≤ 20,000	82 (77.4)	106 (82.2)	106 (86.2)	101 (89.4)	395 (83.9)	0.085
> 20,000	24 (22.6)	23 (17.8)	17 (13.8)	12 (10.6)	76 (16.1)	
Any medical comorbidity^a,c^						
Yes	28 (24.3)	31 (19.6)	31 (18.0)	30 (18.0)	120 (19.6)	0.532
No	87 (75.7)	127 (80.4)	141 (82.0)	137 (82.0)	492 (80.4)	
Regular smoking^d^						
Yes	11 (9.6)	15 (9.5)	19 (10.9)	18 (10.7)	63 (10.2)	0.964
No	104 (90.4)	143 (90.5)	155 (89.1)	150 (89.3)	552 (89.8)	
Regular alcoholic beverage drinking^e^						
Yes	2 (1.7)	6 (3.8)	12 (6.9)	10 (6.0)	30 (4.9)	0.188
No	113 (98.3)	152 (96.2)	162 (93.1)	158 (94.0)	585 (95.1)	
Any symptom during enrollment^f^						
Yes	50 (43.9)	32 (20.4)	11 (6.4)	18 (10.9)	111 (18.3)	** < 0.001**
No	64 (56.1)	125 (79.6)	161 (93.6)	147 (89.1)	497 (81.7)	
Household size^a^						
≤ 4 persons	33 (29.5)	74 (46.8)	80 (46.2)	86 (51.5)	273 (44.8)	**0.003**
> 4 persons	79 (70.5)	84 (53.2)	93 (53.8)	81 (48.5)	337 (55.2)	
Recent exposure to any person with respiratory symptoms^a^						
Yes	22 (19.3)	16 (10.3)	6 (3.4)	1 (0.6)	45 (7.4)	** < 0.001**
No	92 (80.7)	140 (88.6)	168 (96.6)	165 (99.4)	565 (92.6)	
Recent exposure to any person with suspected or confirmed COVID-19^a^						
Yes	5 (4.5)	4 (2.5)	6 (3.5)	0	15 (2.5)	0.081
No	107 (95.5)	153 (97.5)	167 (96.5)	165 (100)	592 (97.5)	
Living with person with COVID-19 signs and symptoms^f^						
Yes	20 (17.4)	8 (5.1)	3 (1.7)	0	31 (5.1)	** < 0.001**
No	95 (82.6)	149 (94.9)	171 (98.3)	166 (100)	581 (94.9)	
Regular mask wearing^a^						
At home and in public	6 (5.2)	8 (5.1)	7 (4.0)	0	21 (3.4)	0.059
In public	108 (93.9)	149 (94.9)	166 (96.0)	165 (98.8)	588 (96.1)	
No	1 (0.9)	0	0	2 (1.2)	3 (0.5)	
Social distancing at home^a^						
Yes	37 (32.5)	80 (50.6)	55 (32.0)	73 (43.7)	245 (39.8)	**0.001**
No	77 (67.5)	78 (49.4)	117 (68.0)	94 (56.3)	366 (59.5)	
Handwashing upon return from outdoors^a^						
Always	104 (90.4)	157 (99.4)	167 (96.0)	162 (97.6)	590 (96.2)	**0.005**
Sometimes	9 (7.8)	0	6 (3.4)	2 (1.2)	17 (2.8)	
Rarely	2 (1.7)	1 (0.6)	1 (0.6)	2 (1.2)	6 (1.0)	
Handwashing upon food consumption						
Always	108 (93.9)	157 (99.4)	167 (96.0)	163 (97.0)	595 (96.7)	0.115
Sometimes	4 (3.5)	0	6 (3.4)	2 (1.2)	12 (2.0)	
Rarely	3 (2.6)	1 (0.6)	1 (0.6)	3 (1.8)	8 (1.3)	
Handwashing with toilet use						
Always	111 (96.5)	157 (99.4)	168 (96.6)	161 (95.8)	597 (97.1)	0.232
Sometimes	4 (3.5)	0	5 (2.9)	4 (2.4)	13 (2.1)	
Rarely	0	1 (0.6)	1 (0.6)	3 (1.8)	5 (0.8)	

P values in bold indicate statistically significant baseline characteristic differences across four data collection periods

^a^Missing data, *n*(%): education—5 (0.8), occupation—8 (1.3), frontline status—26 (4.2), household income—144 (23.4), household size—5 (0.8), comorbidity—3 (0.5), exposure to person with respiratory symptom—5 (0.8), exposure to confirmed or suspected COVID-19 case—8 (1.3), mask wearing—3 (0.5), social distancing—4 (0.7), handwashing upon return—2 (0.3)

^b^Includes healthcare workers, uniformed personnel, sanitary personnel, drivers, delivery and logistics personnel, food production and grocery staff, manual laborers, security personnel, formal and informal vendors, wellness staff, and government workers in justice, security, transport, and social protection sectors

^c^Includes hypertension, diabetes mellitus, bronchial asthma, history of tuberculosis, heart disease, cancer, immunodeficiencies including HIV, cerebrovascular disease, chronic obstructive pulmonary disease, chronic kidney disease, chronic liver disease, hematological conditions, and history of organ and/or bone marrow transplant

^d^Includes participants who reported smoking at least a few days per week

^e^Includes participants who reported drinking alcoholic beverage at least a few days per week

^f^Includes cough, sore throat, runny nose, shortness of breath, loss or change in taste, loss or change in smell, fever, chills, headache, fatigue, muscle pain, joint pain, nausea, and vomiting

At least one comorbid medical condition was present in 120 (19.5%) participants including hypertension (11.2%), diabetes mellitus (3.1%), bronchial asthma (2.6%), cerebrovascular disease (1.8%), and chronic obstructive pulmonary disease (1.0%). Twenty-nine (4.7%) participants reported having two or more comorbidities. Regular smoking at least a few days per week was reported by 63 (10.2%) participants including two (3.5%) of 57 child participants. Moreover, regular alcoholic beverage drinking at least a few days per week was reported by 30 (4.9%) participants including two (3.5%) of 57 child participants.

Across four enrollment periods, the distribution of participant sex, age, frontline work status, monthly household income, presence of medical comorbidity, regular smoking, and regular drinking status was similar. Reporting of regular mask wearing in public and handwashing upon food consumption and with toilet use was high across all enrollment periods. Moreover, reporting of recent exposure to any person with suspected or confirmed COVID-19 was low (range 0–4.5%).

The following differences in participant characteristics across periods were observed. More participants in the first enrollment period reported respiratory symptoms (*p* < 0.001), were from larger households (*p* = 0.003), experienced recent exposure to any person with respiratory symptoms (*p* < 0.001), and resided with someone with COVID-19 signs and symptoms (*p* < 0.001). More participants in the second enrollment period reported practicing social distancing (at least one meter) at home (*p* = 0.001) and regular handwashing upon return from outdoors (*p* = 0.005). More participants in the third enrollment period completed tertiary education (*p* = 0.001), and more cleaning services staff were included in the last enrollment period (*p* = 0.001).

Almost a fifth of total participants were symptomatic (*n* = 111, 18.3%), most of whom were enrolled in the first enrollment period (*n* = 50, 43.9%). The most frequently reported symptoms were similar across periods and included runny nose (*n* = 40, 6.5%), cough (*n* = 36, 5.9%), headache (*n* = 33, 5.4%), and sore throat (*n* = 22, 3.6%). Other symptoms such as fever, chills, general malaise, fatigue, loss of smell, loss of taste, and joint pains were most frequently reported in the first enrollment period.

### SARS-CoV-2 seroprevalence and PCR positivity

**Fig. 2:**
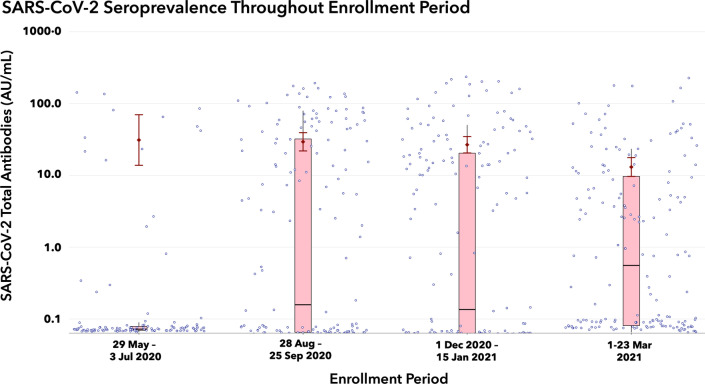
Distribution of SARS-CoV-2 total antibodies across four enrollment periods (*y*-axis at log base 10 scale). Blue circles indicate individual data points from all participants. Pink boxplots show the median [IQR] values of total antibodies per data collection period for all participants. Dark red error bars indicate the geometric mean concentrations and 95% confidence intervals of total antibody among seropositive individuals only

Seroprevalence during the first enrollment period (May–July 2020) was 11.3%. This was significantly smaller than the seroprevalence in the second (46.8%) enrollment period (August–September 2020, *p* < 0.001). Thereafter, seroprevalence was similar between the succeeding third (46.0%) and fourth (44.6%) periods. Among seropositive participants, geometric mean concentrations of total antibodies were comparable throughout the first to third periods. However, total antibody concentration in the third period was significantly higher than that observed in the fourth period (Table 2, Fig. 2).

**Table 2:** SARS-CoV-2 PCR and Antibody Positivity stratified by data collection period

	29 May 2020 to 3 Jul 2020 (*n* = 115)	28 Aug 2020 to 25 Sep 2020 (*n* = 158)	1 Dec 2020 to 15 Jan 2021 (*n* = 174)	1 Mar 2021 to 23 Mar 2021 (*n* = 168)	*p* value
SARS-CoV-2 PCR					
Positive, *N*(%)	9 (7.8)	10 (6.3)	5 (2.9)	16 (9.5)	0.085
95% confidence interval	3.6–14.3%	3.1–11.3%	1–6.6%	5.5–15.0%	
SARS-CoV-2 total antibodies					
Positive, *N*(%)	13 (11.3)	74 (46.8)	80 (46.0)	75 (44.6)	** < 0.001**
95% confidence interval	6.5–18.6%	38.9–54.9%	38.4–53.7%	37.0–52.5%	
Geometric mean, 95% CI (AU/mL)^a^	31.1 (13.8, 70.0)	29.4 (21.9, 39.4)	26.7 (20.6, 34.8)	13.1 (9.7, 17.8)	

*p* value in bold indicates statistically significant outcome difference across four data collection periods

^a^Geometric mean concentrations are calculated among participants who tested positive on SARS-CoV-2 total antibodies

Infection rate (PCR positivity) was similar in each period (range 2.9–9.5%, Table 2), and there was no apparent change pattern. Presence of any respiratory symptom was associated with PCR positivity (*p* < 0.001).

### Characteristics of seropositive and infected individuals

In unadjusted analyses, the odds of seropositivity were greater for frontline workers attending ABC (LRT *p* = 0.050) and lower for clinic attendees with post-secondary education (LRT *p* = 0.001), those with monthly household income greater than 20,000 pesos (LRT *p* = 0.002), or those with any medical comorbidity (LRT *p* = 0.310) (Table 3). Clinic attendees enrolled during the second, third, or fourth periods had more than six times greater odds of seropositivity compared to those enrolled in the first period (LRT *p* < 0.001).

**Table 3:** Association between baseline characteristics and PCR positivity and seropositivity: univariable logistic regression

Variables	Total	PCR positive			Seropositive		
		*n* (%)	OR (95% CI)	*p* value	*n* (%)	OR (95% CI)	*p* value
Enrollment period							
First	115	9 (7.8)	Reference	**0.064**	13 (11.3)	Reference	** < 0.001**
Second	158	10 (6.3)	0.80 (0.31, 2.03)		74 (46.8)	6.91 (3.59, 13.32)	
Third	174	5 (2.9)	0.35 (0.11, 1.07)		80 (46.0)	6.68 (3.49, 12.79)	
Fourth	168	16 (9.5)	1.24 (0.53, 2.91)		75 (44.6)	6.32 (3.30, 12.15)	
Sex							
Female	342	20 (5.8)	Reference	0.462	140 (40.9)	Reference	0.367
Male	273	20 (7.3)	1.27 (0.67, 2.42)		102 (37.4)	0.86 (0.62, 1.19)	
Age							
Below 18	57	7 (12.3)	2.47 (0.98, 6.20)	0.169	24 (42.1)	1.08 (0.61, 1.90)	0.716
18–39	335	18 (5.4)	Reference		135 (40.3)	Reference	
40–49	114	8 (7.0)	1.32 (0.56, 3.15)		44 (38.6)	0.93 (0.60, 1.44)	
50–59	80	3 (3.8)	0.69 (0.20, 2.39)		31 (38.8)	0.94 (0.57, 1.55)	
60 and above	29	4 (13.8)	2.82 (0.89, 8.97)		8 (27.6)	0.56 (0.24, 1.31)	
Post-secondary education^a^							
Yes	416	23 (5.5)	0.65 (0.34, 1.26)	0.210	143 (34.4)	0.53 (0.38, 0.76)	**0.001**
No	194	16 (8.2)	Reference		96 (49.5)	Reference	
Frontline worker^a^							
Yes	205	20 (9.8)	1.97 (1.03, 3.75)	**0.041**	94 (45.9)	1.41 (1.00, 1.99)	**0.050**
No	384	20 (5.2)	Reference		144 (37.5)	Reference	
Monthly household income (PHP)^a^							
> 20,000	76	5 (6.6)	0.96 (0.36, 2.58)	0.935	17 (30.4)	0.43 (0.24, 0.76)	**0.002**
≤ 20,000	395	27 (6.8)	Reference		159 (40.3)	Reference	
Any medical comorbidity^a^							
Yes	120	7 (5.8)	0.86 (0.37, 2.00)	0.725	37 (30.8)	0.63 (0.41, 0.96)	**0.031**
No	492	33 (6.7)	Reference		204 (41.5)	Reference	
Regular smoking							
Yes	63	3 (4.8)	0.70 (0.21, 2.33)	0.537	24 (38.1)	0.94 (0.55, 1.61)	0.829
No	552	37 (6.7)	Reference		218 (39.5)	Reference	
Regular alcoholic beverage drinking							
Yes	30	2 (6.7)	1.03 (0.24, 4.48)	0.971	16 (53.3)	1.82 (0.87, 3.79)	0.113
No	585	38 (6.5)	Reference		226 (38.6)	Reference	
Household size^a^							
> 4 persons	337	30 (8.9)	2.57 (1.23, 5.36)	**0.008**	140 (41.5)	1.21 (0.87, 1.68)	0.253
≤ 4 persons	273	10 (3.7)	Reference		101 (37.0)	Reference	
Exposure to person with respiratory symptoms^a^							
Yes	45	6 (13.3)	2.48 (0.98, 6.28)	**0.078**	16 (35.6)	0.83 (0.44, 1.57)	0.570
No	565	33 (5.8)	Reference		225 (39.8)	Reference	
Exposure to COVID-19 confirmed or suspected persons^a^							
Yes	15	1 (6.7)	1.01 (0.13, 7.90)	0.990	5 (33.3)	0.76 (0.26, 2.27)	0.624
No	592	39 (6.6)	Reference		234 (39.5)	Reference	
Regular mask wearing^a^							
In public	588	37 (6.3)	Reference	0.327	232 (39.5)	Reference	0.538
At home and in public	21	2 (9.5)	1.57 (0.35, 6.99)		7 (33.3)	0.77 (0.31, 1.93)	
No	3	1 (33.3)	7.45 (0.66, 84.02)		2 (66.7)	3.07 (0.28, 34.04)	
Adherence to social distancing at home^a^							
Yes	245	16 (6.5)	1.00 (0.52, 1.92)	0.990	92 (37.6)	0.88 (0.63, 1.22)	0.433
No	366	24 (6.6)	Reference		149 (40.7)	Reference	
Regular handwashing^a^							
Always	590	39 (6.6)	Reference	0.220	229 (38.8)	Reference	0.686
Sometimes	17	0	–		8 (47.1)	1.40 (0.53, 3.68)	
Rarely	6	1 (16.7)	2.83 (0.32, 24.78)		3 (50.0)	1.58 (0.32, 7.88)	

*p* values in bold indicate statistically significant logistic regression results based on likelihood ratio test

^a^Missing data, *n*(%): post-secondary education—5 (0.8), frontline status—26 (4.2), household income—144 (23.4), comorbidity—3 (0.5), household size—5 (0.8), exposure to symptomatic person—5 (0.8), exposure to confirmed or suspected case—8 (1.3), mask wearing—3 (0.5), social distancing—4 (0.7), handwashing—2 (0.3)

After adjustment for sex, age group, and enrollment period, post-secondary education and frontline work status remained associated with increased seropositivity (LRT *p* < 0.001) (Table 4). Clinic attendees who completed post-secondary education were 58% less likely to be seropositive compared to those who did not attain further education, and frontline workers were 81% more likely to be seropositive compared to non-frontline workers. On adjustment, monthly household income (LRT *p* = 0.228) and presence of medical comorbidity (LRT *p* = 0.266) were no longer associated with seropositivity and were not included in the final model.

**Table 4:** Association between baseline characteristics and PCR Positivity and Seropositivity: final model

Variables	Unadjusted OR (95% CI)	Adjusted OR (95% CI)
SARS-CoV-2 PCR positivity		
Sex		
Male	1.27 (0.67, 2.42)	1.19 (0.60, 2.33)
Age group		
Below 18 years	2.47 (0.98, 6.20)	2.91 (0.99, 7.97)
40–49 years	1.32 (0.56, 3.15)	1.32 (0.52, 3.12)
50–59 years	0.69 (0.20, 2.39)	0.85 (0.19, 2.68)
60 years and above	2.82 (0.89, 8.97)	3.17 (0.83, 9.91)
Enrollment period		
Second	0.80 (0.31, 2.03)	0.74 (0.28, 1.99)
Third	0.35 (0.11, 1.07)	0.34 (0.10, 1.05)
Fourth	1.24 (0.53, 2.91)	1.12 (0.47, 2.86)
Household size		
> 4 members	2.57 (1.23, 5.36)	2.45 (1.18, 5.49)
Frontline work status		
Frontliner	1.97 (1.03, 3.75)	2.27 (1.10, 4.75)
SARS-CoV-2 seropositivity		
Sex		
Male	0.86 (0.62, 1.19)	0.94 (0.61, 1.43)
Age group		
Below 18 years	1.08 (0.61, 1.90)	0.79 (0.31, 1.93)
40–49 years	0.93 (0.60, 1.44)	0.90 (0.52, 1.53)
50–59 years	0.94 (0.57, 1.55)	0.88 (0.47, 1.66)
60 years and above	0.56 (0.24, 1.31)	0.42 (0.14, 1.15)
Enrollment period		
Second	6.91 (3.59, 13.32)	7.62 (3.67, 17.28)
Third	6.68 (3.49, 12.79)	7.28 (3.48, 16.58)
Fourth	6.32 (3.30, 12.15)	6.37 (3.01, 14.64)
Post-secondary education		
Present	0.53 (0.38, 0.76)	0.42 (0.26, 0.67)
Frontline work status		
Frontline worker	1.41 (1.00, 1.99)	1.81 (1.18, 2.80)

In unadjusted analyses, the following characteristics were associated with testing SARS-CoV-2 positive: frontline work (LRT *p* = 0.041), large household size (LRT *p* = 0.008), enrollment period (LRT *p* = 0.064), and exposure to any person with respiratory symptoms (LRT *p* = 0.078) (Table 3). In a final multivariable model, adjusting for sex, age group, and enrollment period, frontline work status and those with a large household size (> 4 persons) remained associated with PCR positivity (LRT *p* = 0.020) (Table 4). Frontline workers were associated with more than twice greater odds of infection compared to non-frontline workers, and those belonging to large households were also associated with more than twice greater odds of PCR positivity compared to members of small households (≤ 4 persons). On adjustment, exposure to any person with respiratory symptoms did not remain associated with PCR positivity (LRT *p* = 0.104); hence, it was not included in the final model.

Age and sex were not associated with seropositivity or infection in univariable or multivariable analyses.

## Discussion

In this study, we performed four repeated cross-sectional surveys to estimate SARS-CoV-2 seroprevalence and infection rate among ABC attendees as a surrogate of the catchment population in Metro Manila over four periods from May 2020 to March 2021.

Our seroprevalence estimates quadrupled between the first and second periods (between May and September 2020) and were comparable in the succeeding periods. The infection-induced seroprevalence in the fourth enrollment period (March 1–23, 2021) of 44.6% (95% CI 37.0–52.5%) among community dwellers was comparable to the estimated 36.0% (95% CI 30.0–38.1%) seroprevalence from healthcare workers with and without direct exposure to COVID-19 positive patients and/or specimens in the same infectious disease referral hospital (March 8–April 24, 2021), prior to vaccine rollout [9].

Repeated seroprevalence estimations conducted in healthy blood donors have shown variable rates of change through time. Dramatic rise in antibody levels from 0% in early 2020 and 27.4% in early 2021 was documented in Jordan [23], and more subtle increase from 0.8% in April 2020 to 6.3% in March 2021 was reported in Canada [24]. The quadrupling of infection-induced seroprevalence over just a 3-month period has not been previously reported even in the UK, which performs large-scale community-level seroprevalence and COVID-19 positivity studies [25]. Similarly, the rapid seroprevalence rise in our study has also not been previously reported in the US, which had the highest number of cumulative COVID-19 cases [26]. Instead, overall infection-induced seroprevalence, analyzed for anti-nucleocapsid (*N*) antibodies which are produced in response to infection but not to vaccination, jumped from 33.5% to 57.7% between December 2021 and February 2022 at the height of B.1.1.529 (Omicron) variant surge in the US [27].

There is scarcity of repeated estimations that document plateauing or decreasing seroprevalence. However, immune response duration depends on individual factors, antigen type, antibody isotype, and assays performed. SARS-CoV-2-specific IgG appears to be most durable and may remain detectable up to 10-month post-infection [28]. Persons with moderate to severe COVID-19 illness also have higher titers of binding and neutralizing antibodies than those with mild disease, with persistence of differences up to 8-month post-infection [29].

We hypothesize the following explanations for the observed rapid rise then plateau in our repeated seroprevalence estimations. Between the first and second data collection periods, the epidemic may have spread rapidly in the communities across Metro Manila. Assuming immune response persisted up to 10 months, the rise in new cases reported in Manila and the Philippines between the first and second enrollment periods may have driven the population-level seropositivity seen throughout the remaining enrollment periods.

Possibly by the second enrollment period, individuals who were living in more crowded conditions and who needed to move across the cities for employment were already infected. That large household size and frontline work status were associated with greater odds of PCR positivity is consistent with current evidence on transmission dynamics in households [30] and at the workplace [31], especially prior to vaccination.

Those who belonged to higher socioeconomic strata may have had greater social capital to limit exposures by working remotely. In our study, we found attainment of post-secondary education to be associated with lesser odds of seropositivity and frontline work to be associated with greater odds of seropositivity. Current evidence on the effect of education on seropositivity is mixed—from no association [32] to being associated with greater odds of seropositivity [33, 34]. The possibility of confounding or effect modification of enrollment period with other unmeasured variables cannot be discounted.

While the last enrollment period coincided with the upward trajectory of the second COVID-19 wave in the country, the duration of our data collection may not have been sufficient to capture the increasing exposures expected with increasing cases.

A potential equilibrium between stable disease transmission and waning immunity may also explain the observed plateau in the seroprevalence estimates. Longitudinal analysis of antibody dynamics suggests that mild and asymptomatic disease are associated with earlier clearance of neutralizing antibodies and IgG compared to severe disease [35]. Most clinic attendees are asymptomatic or mildly symptomatic and may be prone to exhibiting shorter duration of immunity. More infections may be taking place in the community, but its effects on seroprevalence are dampened by the waning immunity. The lower total antibody concentration seen among seropositive individuals in the fourth period compared to the third period may be evidence supporting waning immunity (Fig. 2). However, because our study did not involve longitudinal analysis of specimens from the same individuals, we cannot confirm if the observed antibody levels represent the true effect of change with time.

The comparable seroprevalence estimates between the second to fourth period may have also reflected the dynamics of dominant SARS-CoV-2 variants in the country. Unfortunately, there is no reliable variant epidemiologic surveillance in the Philippines especially in early 2021, preventing us from exploring this hypothesis.

Widespread adherence to non-pharmaceutical interventions, especially among clinic attendees, may also have impeded the further rise in seroprevalence in the latter periods. Assuming individuals maintained their protective practices and had reduced exposures as reported in the third to fourth periods, seroprevalence is expected to plateau. The estimated seroprevalence in our last enrollment period of 44.6% (95% CI 37.0–52.5%) is comparable to the peak seropositivity in England among all adults of 54.7% (95% CrI 49.3–60.5%) prior to vaccination roll out. [36] This suggests that peak population-level COVID-19 infection-induced immunity may be insufficient and highlights the need for vaccination to boost immune response. Reliability of self-reported exposures limits our ability to explore this hypothesis.

Despite the changing incidence of COVID-19 infection nationally during the study (Fig. 1), we did not find significant variation in COVID-19 infection across four periods (2.9–9.5%). This is consistent with our hypothesis that assuming most symptomatic individuals either isolate or consult in dedicated fever and/or respiratory infection clinics, the observed change pattern in PCR positivity across periods will be minimal.

Repeated cross-sectional analysis of SARS-CoV-2 positivity in other contexts is scarce. A population-based nationwide prevalence survey involving 11 rounds of sampling from April 2020 to February 2021 among adults in Estonia found very low COVID-19 prevalence (2% in the last data collection) despite reaching more than 40 times greater number of new confirmed COVID-19 cases per million than the Philippines in the same period [37]. The larger estimated population prevalence in our study may be attributed to our catchment area of Metro Manila, which had the largest concentration of overall reported cases in the Philippines, whereas the prevalence estimates in Estonia were sampled from all over the country.

We also found that reporting of mask wearing in public settings and handwashing upon return from outdoors, with food consumption, and with toilet use was consistently very high (> 90%). Our results are in contrast with the declining trends (range 3–14% reduction) in handwashing practice for COVID-19 prevention observed in ten sub-Saharan African countries across two periods (July 2020 and November 2020) [38].

Our study has several limitations. First, due to resource and logistic constraints as the epidemic progressed, we enrolled a relatively small sample size during individual data collection periods. This prevented us from performing a population-weighted seroprevalence estimation. A larger sample size would have improved precision in estimation of outcomes and increased internal and external validity of a study collecting data at an outpatient clinic with a large catchment area.

Second, the repeated cross-sectional nature of our study introduced variability in some of the characteristics of study participants when stratified by enrollment period. We anticipated these potential differences and addressed them by adding enrollment period a priori to model building. Large-scale longitudinal cohort design would have allowed better characterization of changes in seroprevalence through time and potential associated risk factors.

Third, we made categorizations in selected variables to aid regression analysis. We categorized occupation according to frontline work status based on vaccination priority list; however, we did not have actual work exposure data and did not inquire on individual ability to isolate and/or work remotely.

Fourth, we included children in all our analyses despite presence of age-related variables, such as occupation, education, household income, smoking, and alcoholic beverage use. Some household income data were also available. Fifth, we cannot discount the effect of social desirability and response bias on self-reported variables in our study.

We were not able to perform a dedicated in-house validation study for the Elecsys Anti-SARS-CoV-2 immunoassay due to resource limitations. The published test performance data were based on studies involving symptomatic individuals who tested positive on PCR [16]. In contrast, most of our participants were asymptomatic, which may have led to an underestimation of true seroprevalence. A potential cause of antibody test false positivity is cross-reactivity with other analytes. In the original validation study, 4/792 samples contained cross-reacting analytes including cytomegalovirus, Epstein Barr virus, and systemic lupus erythematosus [16, 17]. There was no cross-reactivity for other coronaviruses.

Finally, this study was not designed as a community-based seroprevalence estimation with random selection of participants. On one hand, the use of a healthcare facility as a study setting may have introduced selection bias favoring enrollment of individuals with good health-seeking behavior and better personal protective practices. This may have led to an underestimation of seroprevalence. On the other hand, because the clinic catered to patients from highly urbanized cities with greater population density and higher transportation connectivity than the rest of the country, our results may have led to overestimation of both seroprevalence and infection rate by enrolling highly exposed individuals. There is uncertainty given these factors. However, we deemed the ABC to be a suitable, informative source of seroprevalence data in Metro Manila, because the clinic attendees came from all over the National Capital Region and were asymptomatic or mildly symptomatic individuals seeking care for non-COVID-19-like illness. Moreover, because the clinic remained operational during lockdowns and due to closure of similar centers across the region, the site was able to capture the varying demographics of catchment population during the enrollment periods. While our results may be reflective of the situation in Metro Manila, they may not be generalizable nationally or to other regions, especially those in other island groups.

## Conclusions

Between May 2020 and March 2021, we found constantly high levels of reported observance of personal protective practices for COVID-19 among SLH ABC attendees. Infection-induced seroprevalence quadrupled between the first and second periods and plateaued thereafter. This may be related to the ability of individuals to limit exposure based on socioeconomic status, such as education and employment. Despite varying disease incidence and stringency index, infection rates were comparable across the four rounds of enrollment. Belonging in a large household and being a frontline worker were associated with greater odds of PCR positivity. Large-scale longitudinal cohort studies would better enable monitoring of seroprevalence, community-level immunity, and associated risk factors.

## Data Availability

Data and materials used in this study may be obtained from the corresponding author based on reasonable request.

## Abbreviations

ABC: Animal bite clinic
ARI: Acute respiratory tract infection
COVID-19: Coronavirus disease
DOH: Department of Health
HCW: Healthcare workers
LRT: Likelihood ratio test
RT-PCR: Real-time polymerase chain reaction
SARS-CoV-2: Severe acute respiratory syndrome coronavirus 2
SLH: San Lazaro Hospital
